# Supplementary material for: Recent Developments in Nanomaterials-Based Drug Delivery and Upgrading Treatment of Cardiovascular Diseases
Source: Int J Mol Sci. 2022 Jan 26;23(3):1404. doi: 10.3390/ijms23031404 (PMC8836006; doi:10.3390/ijms23031404)
Supplement: Supplementary file 1 [file ijms-23-01404-s001.zip › ijms-1485980-supplementary.pdf]

Table S1: FDA and EMA approved lipid nanoformulations, their composition, loaded drug, producing company, and approval year[1-4].

No.	Drug nanoparticle conjugate commercial name	Loaded drug name	Pharmaceutical Company	Clinical Indications	FDA/EMA Approval Year(s)
1	Inflexal® V	Monovalent virosome pools from three influenza strain in a mixture of hemagglutinin and neuraminidase glycoproteins.	Crucell Spain S.A	Used as adjuvanted influenza (flu) Vaccination.	EMA July 1, 2001*
2	Definity®	Perflutren	Dupont Pharmaceuticals Company	Used as an ultrasound contrast agent, it can improve the quality of echocardiograms.	FDA July 31, 2001
3	Copaxone®	Glatiramer acetate	Eva Pharmaceutical Industries LTD	Used to treat relapsing forms of multiple sclerosis	FDA August 15, 2011.
4	SonoVue® LUMASON	Sulphur hexafluoride	Bracco International B.V.	Used as an ultrasound contrast agent.	FDA October 2014<br>EMA March 26, 2001
5	ONPATTRO®, Patisiran-LNP®, ALN-TTR02®	Patisiran Sodium	Alnylam Pharmaceuticals, Inc	Used for the treatment of the polyneuropathy of hereditary transthyretin-mediated amyloidosis in adults.	FDA August 10, 2018<br>EMA August 27, 2018
6	Moderna COVID-19 Vaccine	Synthetic mRNA-1273, encoding stabilized prefusion SARS-CoV-2 spike protein	Moderna Therapeutics Inc.	Induces antibody production against SARS-CoV-2	FDA and EMA 2020 Emergency use authorization

7	Pfizer-BioNTech COVID-19 Vaccine	Nucleoside modified mRNA BNT162b2, encoding SARS-CoV-2 spike protein	Pfizer-BioNTech COVID-19 Vaccine Comirnaty®	Induces antibody production against SARS-CoV-2	FDA and EMA 2021

*Discontinued

Table S2: FDA and EMA approved liposome nanoformulations, their composition, loaded drug, producing company, and approval year[1-3].

No.	Drug nanoparticle conjugate commercial name	Loaded drug name	Pharmaceutical Company	Clinical Indications	FDA Approval Year(s)
1	Doxil®/CaelyxTM	Doxorubicin	Janssen	Used to treat the followings: Breast, ovarian cancer, and HIV-related Kaposi's sarcoma Myeloma.	FDA November 17, 1995<br>May 17, 2007<br><br>EMA June 20, 1996
2	Abelcet®	Amphotericin B	Leadiant Biosciences, Inc.	Fungal infections	FDA November 20, 1995
3	DaunoXome®	Daunorubicin	Galen	Used to treat HIV-related Kaposi's sarcoma	FDA April 8, 1996<br>EMA July 26, 2004
4	AmBisome®	Amphotericin B	Gilead Sciences	Used to treat Fungal Infection	FDA August 11, 1997<br>EMA August 28, 2006
5	DepoCyt©	Cytarabine	DepoTech Corporation/ Pacira Pharmaceuticals	Used for the intrathecal treatment of lymphomatous meningitis.	FDA August 1, 1999<br>EMA November 7, 2001
6	Curosurf®/Poractantalpha	Pulmonary surfactant (Protein SP-B and SP-C)V	Chiesi Farmaceutici	Used to treat Respiratory Distress Syndrome (RDS) in premature infants with an endogenous pulmonary surfactant deficiency.	FDA November 18, 1999
7	Myocet®	Doxorubicin hydrochloride (HCl), and an anthracycline cytotoxic agent	Teva UK	Used in combination with the anti-cancer cyclophosphamide for the treatment of metastatic breast cancer in adult females who are > 18 years.	FDA 2000<br>EMA July 7, 2000
8	Visudyne®	Verteporfin	Bausch & Lomb Inc	Used to treat certain severe eye conditions (e.g., macular degeneration, decreased	FDA April 12, 2000<br>May 29, 2002<br>EMA July 27, 2000

				vision, pathologic myopia, ocular histoplasmosis	
9	Mepact®	Mifamurtide	Takeda Oncology Company (Millennium)	Used to treat high-grade non-metastatic osteosarcoma and myosarcoma. Patient age limit is 2 to 30 years old.	FDA 2001<br>EMA March 6, 2009
10	Propofol®/ Diprivan®	Propofol	AstraZeneca LP	General anesthetic agent used to induce and maintain anesthesia. It is also used to sedate a patient who is under critical care and needs a mechanical ventilator (breathing machine)	FDA February 23, 2001<br>EMA September 24, 2004.<br>July 9,2007.<br>September 16, 2011
11	DepoDur®	Morphine Sulfate	SkyePharma PLC and Endo Pharmaceuticals	Treatment for Post-Surgical Pain Relief	FDA May 18, 2004**<br>EMA May 18, 2004
12	Exparel	Bupivacaine	Pacira	Anesthetic	FDA and EMA 2011
13	Marqibo®	Vincristine.<br>Also known as leurocristine and marketed as brandname.	Talon Therapeutics, Inc	Philadelphia chromosome negative (Ph-) acute lymphoblastic leukemia (ALL).	FDA and EMA August 9, 2012
14	Lipodox	Doxorubicin	Sun Pharma Advanced Research Company	Breast neoplasms	FDA and EMA 2013
15	Onivyde®	Irinotecan	Merrimack	Pancreatic cancer	FDA October 22, 2015<br>EMA December 9, 2011
16	Liposomal doxorubicin	Doxorubicin	Taiwan Liposome Company	Breast neoplasms	FDA and EMA 2017
17	Nocita®	Bupivacaine	Aratana Therapeutics, Inc.	Anesthetic	FDA and EMA 2017
18	VYXEOS®-CPX-351®	Cytarabine and Daunorubicin	Jazz Pharmaceuticals	Used to treat acute myeloid leukemia (AML).	FDA August 3, 2017<br>EMA July 31, 2018

19	ArikayceTM	Amikacin	PARI Pharma GmbH	Mycobacterium avium complex lung disease	FDA and EMA 2018
20	LipoplatinTM /NanoplatinTM	Cisplatin	Regulon	Pancreatic cancer<br>Lung cancer	FDA and EMA 2018

*Discontinued

*Discontinued ** DepoDur is discontinued, but generic versions may be available.

Table S3: FDA and EMA approved polymer nanoformulations, their composition, loaded drug, producing company, and approval year[1-3].

No.	Drug nanoparticle conjugate commercial name	Loaded drug name	Pharmaceutical Company	Clinical Indications	FDA Approval Year(s)
1	Adagen®/pegademase bovine	Adenosine deaminase enzyme	Sigma-Tau Pharmaceuticals, Inc	Used to treat severe combined immunodeficiency disease (SCID) associated with a deficiency of adenosine deaminase.	FDA March 20, 1990<br>EMA March 20, 1990
2	Copaxone®/Glatopa	Glatiramer Acetate	Teva Pharmaceutical Industries LTD	Used to treat multiple sclerosis	FDA December 20, 1996<br>April 16, 2015
3	Oncaspar ®	L-Asparaginase	Enzon Pharmaceuticals	Used to treat acute lymphocytic leukemia (ALL), especially in patients who are allergic to L-asparaginase.	FDA February 1, 1994<br>EMA January 14, 2016
4	Renagel®<br>Renvela®	Sevelamer hydrochloride or sevelamer carbonate	SANOFI	Hyperphosphatemia in patients on dialysis and chronic renal disease	FDA July 12, 2000<br>October 19, 2007<br>EMA January, 28 2000
5	PegIntron®	Interferon-alfa (INF-a2b) protein	Merck	Hepatitis C	FDA August 7, 2001<br>EMA May 24, 2000*
6	Eligard®	Leuprolide Acetate	Tolmar	Advanced prostate cancer	FDA January 23, 2002<br>EMA May 14, 2020
7	Neulasta®/pegfilgrastim	Filgrastim, granulocyte colony-stimulating factor (G-CSF) protein memetic.	Amgen	Neutropenia and leukopenia caused by chemotherapy.	FDA March 1, 2002<br>EMA August 22, 2002
8	Pegasys®	Interferon alfa (INF-a2a)	Genentech	Hepatitis C<br>Hepatitis C	FDA October 15, 2002<br>October 29, 2004

					EMA June 20, 2002
9	Somavert®/pegvisomant	Growth Hormone Receptor (GHR) antagonist. Visomant	Pfizer	Used to treat acromegaly	FDA March 25, 2003<br>EMA November 13, 2002
10	Macugen®/ Pegaptanib	Anti-vascular endothelial growth factor (VEGF) aptamer. Aptanib.	Bausch & Lomb Inc	Treatment for Neovascular (Wet) Age-Related Macular Degeneration (AMD).	FDA December 17, 2004<br>EMA January 2006*
11	Mircera®/Methoxy	Synthetic epoetin beta-protein erythrocyte stimulating agent (ESA)	Hoffmann-La Roche Ltd	Anemia in adult with chronic renal failure (CRF)	FDA November 14, 2007<br>EMA July 20, 2007
12	Cimzia®/certolizumab pegol	Certolizumab fragment	UCB, Inc	Crohn's disease<br>Rheumatoid arthritis<br>Psoriatic arthritis,<br>Active ankylosing spondylitis<br>Plaque psoriasis,<br>Non-radiographic axial spondyloarthritis.	FDA April 22, 2008<br>May 13, 2009<br>Sep 30, 2013<br>Oct 18, 2013<br>May 28, 2018<br>March 29, 2019<br>EMA October 1, 2009
13	Krystexxa®/Pegloticase	porcine-likeuricase	Horizon Pharma Rheumatology LLC	Chronic gout	FDA September 15, 2010<br>EMA January 8, 2013
14	Plegridy®	IFNbeta-1a (IFN-β1a)	Biogen	Multiple Sclerosis	FDA August 18, 2014<br>EMA July 18, 2014
15	ADYNOVATE®	Antihemophilic Factor (VIII)	Baxalta	Hemophilia A	FDA November 13, 2015<br>EMA January 8, 2018

*Discontinued

Table S4: FDA and EMA approved metal nanoformulations, their composition, loaded drug, producing company, and approval year[1-3].

No.	Drug nanoparticle conjugate commercial name	Loaded drug name	Pharmaceutical Company	Clinical Indications	FDA Approval Year(s)
1	INFeD®	Iron	Pharmacosmos	Used to treat chronic kidney failure with iron-deficiency anemia	FDA August 11, 1992
2	DexIron®/Dexferrum®	Iron	Sanofi Aventis	Used to treat chronic kidney failure with iron-deficiency anemia	FDA February 23, 1996
3	Feridex®/Endorem®	SPION coated with dextran	AMAG Pharmaceuticals	Magnetic Resonance Imaging (MRI) contrast agent.	FDA August 30, 1996*
4	GastroMARKTM/umirem®	SPION coated with silicon	AMAG pharmaceuticals	Magnetic Resonance Imaging (MRI) contrast agent.	FDA December 6, 1996<br>March 1, 2009
5	Ferrlecit®	Sodium ferric ion gluconate complex	Sanofi-Aventis	Used to treat iron deficiency anemia in patients (>6 years) with chronic kidney disease who are receiving hemodialysis and epoetin supplement therapy.	FDA February 18, 1999<br>EMA April 1, 2011
6	Venofer®	Iron sucrose	Luitpold Pharmaceuticals, Inc.	Used to treat chronic kidney failure with iron deficiency anemia.	FDA November 7, 2000
7	Resovist®	Super-paramagnetic Iron Oxide Nanoparticles (SPION)		Used as a contrast agent for liver magnetic resonance imaging (MRI).	EMA August 3, 2001*.
8	Ferumoxtran-10®/Combidex®	Super-paramagnetic Iron Oxide	AMAG Pharmaceuticals, Inc.	Used as a functional molecular imaging agent in conjunction with magnetic resonance imaging (MRI) to aid in the	FDA March 2005<br>EMA March 26, 2019 (Approved in the Netherlands only)

		Nanoparticles (SPION)		differentiation of cancerous from non-cancerous lymph nodes.	
9	FerahemeTM/ferumoxytol	Ferumoxytol Superparamagnetic Iron Oxide Nanoparticles (SPION)	AMAG Pharmaceuticals, Inc	Used to treat chronic kidney failure with iron deficiency anemia.	FDA June 30, 2009<br>EMA July 13, 2015
10	Nanotherm®	Iron-oxide magnetite	MagForce	Used for the treatment of solid tumors.<br>Glioblastoma	FDA August 11, 2010<br>EMA May 2013
11	Rienso®	Iron-carbohydrate complex (Ferumoxytol)	Takeda Pharma A/S.	Used to treat patients with iron-deficiency anemia associated with chronic kidney disease.	EMA June 15, 2012.
12	Ferinject®/Injectafer®	Ferric carboxymaltose	Vifor Pharma	Used to treat iron deficiency conditions	FDA July 2013<br>EMA September 13, 2013.
13	Ferrisat®/Cosmofer®	Iron(III) isomaltoside dextran complex	Pharmacosmos	Used to treat iron-deficiency anemia.	EMA September 16, 2013.
14	Monofer®	Ferric derisomaltose (Irron(III) isomaltoside)	Pharmacosmos	Used to treat iron-deficiency anemia.	FDA 2020<br>EMA December 22, 2009.<br>September 13, 2013.

*Discontinued

Table S5: FDA and EMA approved nanocrystals nanoformulations their composition, loaded drug, producing company, and approval year[1-3].

No.	Drug nanoparticle conjugate commercial name	Loaded drug name	Pharmaceutical Company	Clinical Indications	FDA Approval Year(s)
1	Epaxal®	Purified hepatitis A virus (HAV) virions, of the RG-SB of the A strain.	Crucell	Used to vaccinate against hepatitis A	EMA December 14, 1999.<br>April 25, 2007.*
2	Rapamune®	Sirolimus	Wyeth-Ayerst Research/ Pfizer Irland Pharmaceuticals, IE.	Immunosuppressant after kidney transplant.<br>Used to treat a rare lung disorder called lymphangioleiomyomatosis	FDA August 25, 2000<br>May 29, 2015<br>EMA March 14, 2001
3	Zevalin®	Monoclonal antibody ibritumomab bound to tiuxetan	Bayer pharma	Used to treat non-Hodgkin's lymphoma (NHL)	FDA February 19, 2002<br>EMA January 16, 2004
4	Avinza®	Morphine Sulfate	Pfizer Inc	Mental stimuli. Used to treat moderate to severe pain	FDA March 20, 2002*<br>EMA December 3, 2010
5	Ritalin LA®	Methylphenidate hydrochloride. Central nervous system stimuli.	Novartis	Mental stimuli. Attention-Deficit/Hyperactivity Disorder	FDA May 6, 2002<br>EMA May 27, 2009
6	Zanaflex®	Tizanidine HCL	Covis Pharma Bv	Used to treat spasticity associated with diseases like multiple sclerosis and spinal cord injuries by temporarily relaxing muscle tone.	FDA August 29, 2002
7	Emend®	Aprepitant, a P/neurokinin 1 (NK1) receptor antagonist.	Merck	Antiemetic drug. Chemotherapy-Induced Nausea and Vomiting (CINV)	FDA March 26, 2003<br>EMA November 11, 2003

8	OsSatura®	Bone mineral mimetic mixture (Hydroxyapatite (HA))	IsoTis Orthobiologics	Bone Void Filler/Bone Graft Substitute	FDA June 3, 2003
9	Vitoss®	Bone mineral mimetic mixture (calcium phosphate)	Stryker	Resorbable Synthetic Bone Void Filler/Bone Graft Substitute	FDA August 29, 2003
10	Zypadhera®	Olanzapine	Lilly Pharma	Used to improve symptoms of patients with schizophrenia	FDA March 29,2004<br>EMA November 19, 2008
11	Tricor®/Lipanthyl®/Lipidil®	Fenofibrate	Recipharm, FR	Used to treat hypercholesterolemia and hypertriglyceridemia.	FDA May 11, 2004<br>EMA February 28, 2011
12	Ostim®	Nanocrystalline paste consists of a suspension of pure hydroxyapatite (HA) in water.	Heraeus kulzer	Bone substitute	FDA July 13, 2004
13	Tricor®	Fenofibrate	Lupin Atlantis	Used for the treatment of different types of hypercholesterolemia and hypertriglyceridemia.	FDA November 5, 2004<br>EMA April 12, 2018
14	NanOss®	Osteoconductive, resorbable, calcium phosphate, and Hydroxyapatite (HA) pellets implant.	RTI Surgical, Inc	Bone void fillers and bone substitutes.	FDA February 03, 2005
15	Focalin XR®	Dexmethylphenidate HCL	Novartis Pharmaceuticals.	Mental stimuli. Central nervous system stimuli used for the treatment of the Attention-	FDA May 26, 2005<br>November 12, 2008

				Deficit/Hyperactivity Disorder (ADHD)	
16	MegaceES®	Progesterone memetics (Megestrol Acetate)	Par Pharmaceutical Inc.	Anti-anorexic. Used to treat loss of appetite and wasting syndrome in people with acquired immunodeficiency syndrome (AIDS)	FDA July 7, 2005
17	Invega®Sustenna	Paliperidone palmitate	Janssen Pharmaceuticals, Inc	Used for the following mental/mood disorders:<br>Schizophrenia<br>Schizoaffective disorder	FDA July 31, 2009<br>November 13, 2014<br>EMA June 25, 2007
18	Xeplion®	Paliperidone	Janssen Pharmaceuticals NV.	Used to maintenance treatment of patients with schizophrenia.	FDA July 31, 2009<br>EMA March 4, 2011
19	EquivaBone®	Amorphous and crystalline mixture of Calcium phosphate sets, the human demineralized bone matrix (DBM), the synthetic carboxymethyl cellulose (CMC) and hydroxyapatite (HA) crystals	Zimmer Biomet	Used as bone Graft Substitute.	FDA September 18, 2009
20	Ryanodex®	Dantrolene sodium	Eagle pharmaceuticals	Malignant hyperthermia	FDA July 22, 2014<br>EMA December 12, 2016
21	NBTXR3®, Hensify®	Nanobiotix	Nanobiotix	Used to treat locally advanced soft tissue sarcoma, carcinoma.	FDA 2017<br>EMA April 4, 2019

*Discontinued

Table S6: FDA and EMA approved protein nanoformulations their composition, loaded drug, producing company, and approval year[1-3].

No.	Drug nanoparticle conjugate commercial name	Loaded drug name	Pharmaceutical Company	Clinical Indications	FDA Approval Year(s)
1	Optison®	Perflutren	GH Healthcare	Used as an ultrasound contrast agent to improve echocardiograms.	FDA December 31, 1997
2	Ontak®	Engineered protein combining diphtheria and L-2 toxins	Eisai Inc	Used for the treatment of Cutaneous T-cell lymphoma (CTCL)	FDA February 5, 1999*
3	PegIntron®	Interferon alfa-2b (IFN-2b)	Schering-Plough	Used to treat chronic hepatitis C in adults.	FDA August 7, 2001<br>EMA May 25, 2000
4	Neulasta®	Filgrastim	Amgen Technology (Ireland)	Used to treat leukopenia caused by cancer chemotherapy.	FDA January 31, 2002<br>EMA August 22, 2002.<br>April 26, 2019.
5	Pegasys®	Interferon alfa-2a (IFN-2a)	Roche Pharma	Used to treat chronic hepatitis B in adults and to treat hepatitis C in adults as well as children who are at least 5 years old.	FDA 2002<br>EMA June 20, 2002
6	Somavert®	Human growth hormone (HGH) antagonist Visomant	Pfizer Manufacturing.UK	Used to treat acromegaly	FDA March 25, 2003<br>EMA November 13, 2002
7	Abraxane®/ABI-007	Paclitaxel	Abraxis BioScience/ Celgene	Breast Cancer<br>Non-small Cell Lung Cancer	FDA January 07, 2005<br>October 12, 2012<br>Sep 6, 2013<br>Mar 8, 2019

				Late-Stage Pancreatic Cancer<br>To be used in combination with Tecentriq for adults with To be triple-negative breast cancer (TNBC) with tumor expressing PD-L1	EMA January 10, 2008
8	Mircera®	Methoxy polyethylene glycol-epoetin beta	Roche Pharma	Used to treat anemia caused by cancer chemotherapy.	EMA July 20, 2007
9	Cimzia®	Certolizumab (PEG-anti-TNFFab)	UCB Pharma SA	Used for certain inflammatory conditions (such as rheumatoid arthritis, psoriatic arthritis, ankylosing spondylitis, plaque psoriasis).	FDA May 13, 2009<br>EMA October 1, 2009

*Discontinued

Table S7: FDA and EMA approved other nanoformulations their composition, loaded drug, producing company, and approval year[1-3].

No.	Nanoformulation	Drug nanoparticle conjugate commercial name	Loaded drug name	Pharmaceutical Company	Clinical Indications	FDA/EMA Approval Year(s)
1	Micelles	EstrasorbTM	Estradiol	Novavax Inc	Menopause hormone therapy. Hormone therapy to relieve menopause symptoms	FDA September 10, 2003
2	Nanoemulsions PEgylated	Oncaspar®	L-asparaginase	Sigma-Tau Pharmaceuticals, Inc.	Used to treat patients with acute lymphocytic leukemia (ALL) who are allergic to L-asparaginase.	FDA January January 14 14, 2016
3	Nanoemulsions PEgylated	Renagel® Renvela®	sevelamer	Genzyme Ireland Ltd	Dialysis, hyperphosphatemia. Used to control phosphorus levels in chronic kidney failure patients.	EMA January 28, 2000 June 9, 2009
4	Nanoemulsions PEgylated	Norvir®	Copovidone, sorbitan monolaurate, and colloidal silicon dioxide.	Aesica Queenborough Ltd	Immunosuppressants	EMA August 26, 1996

*Discontinued

References:

1. Siyoung A. Lim AC, Madelynn Tung, Eun Ji Chung. Clinical progress of nanomedicine-based RNA therapies. *<div style="">Bioactive Materials*. 2021.
2. Agency EM. https://www.ema.europa.eu/en, 2019.
3. Administration USAFaD. https://www.fda.gov 2019.
4. Thi TTH SE, Lee JS, Nguyen DH, Park KD, Truong NP. Lipid-Based Nanoparticles in the Clinic and Clinical Trials: From Cancer Nanomedicine to COVID-19 Vaccines. *Vaccines (Basel)*. 2021; 9: 359.
